# "Alcoholic" Diseases

**Published:** 1889-01-05

**Authors:** 


### Within the Wards.

### "ALCOHOLIC" DISEASES.

A discussion of great importance took place at the Pathological Society of London a week or two ago. The subject was the "material changes" produced in various tissues and parts of the human body by the use of alcohol in excess. It is obvious that here there is little room for mere speculation. Whatever may be thought of mental, moral, or even of "health" changes, and however contrary may be the opinions expressed, there can be no doubt or uncertainty when "material changes" are under consideration. Dr. Payne, who opened the discussion, and referred specially to changes in the liver, brain, and other organs, which are familiar to all medical men, formulated three general conclusions of great significance. He considers (I) that alcohol checks oxidation, and favours the accumulation of fat. (II.) That it acts as a functional stimulus, exciting organs to abnormal action. (III.) That it acts as a tissue poison, destroying the vitality of some tissue elements, and setting up inflammation in others. Dr. George Harley holds: (I.) That the action of alcohol on the tissues is purely chemical. (II.) That alcohol prevents oxidation. (III.) That many curious results have followed upon the artificial injection of alcohol into animals, among the rest "diabetes." (IV.) That nevertheless alcohol is sometimes a valuable medicine in disease, and that it is a food. These pronouncements may be taken as indicating, not only the state of professional opinion on the subject, but also the condition of positive knowledge. There are undoubtedly certain changes produced in organs of the body, which can be seen with the eye and handled with the hands. These changes are of such a nature that, when they reach a certain stage, death is inevitable. Alcohol undoubtedly kills. But short of that it produces measureless harm. It "checks oxidation," say both Dr. Payne and Dr. Harley. That is, it produces an initial stage of suffocation; it diminishes the usefulness of the lungs; it prevents the proper destruction and removal of the waste tissues of the body; it thereby blocks the excreting organs, and diminishes their activity; it thereby also makes the blood impure, and consequently diminishes the nutritive value of it. It turns the blood into a bad builder, and a bad scavenger as well. This is only when it is taken in excess. A very little is excess for some people. Nevertheless, say the experts, it is a medicine and a food also. If anyone laughs at that, let him consider that so common a thing as table salt is undoubtedly a food; but it is as certainly a medicine, and as unequivocally a poison, in sufficient quantities. All persons should distinctly admit to themselves that alcohol is a poison, and a dangerous poison. It has its uses, and these uses are such that ordinary intelligence may safely be trusted to understand them. But whoever uses alcohol should never forget that he is using a substance which is only safe when the c'earest intelligence and the firmest will are brought into daily exercise. Here, as in other departments of life, sense and conscience are alone to be trusted.

